# The NS1 protein of contemporary West African Zika virus potentiates viral replication and reduces innate immune activation

**DOI:** 10.1371/journal.pntd.0012146

**Published:** 2024-08-23

**Authors:** Dana Machmouchi, Marie-Pierre Courageot, Eva Ogire, Lars Redecke, Alain Kohl, Philippe Desprès, Marjolaine Roche

**Affiliations:** 1 Processus Infectieux en Milieu Insulaire Tropical (PIMIT), Université de La Réunion, INSERM U1187, CNRS 9192, IRD 249, Plateforme Technologique CYROI, Sainte-Clotilde, La Réunion, France; 2 UR7506-BioSpect, Université de Reims Champagne-Ardennes, Reims, France; 3 University of Luebeck, Institute of Biochemistry, Luebeck, Germany; 4 Deutsches Elektronen Synchrotron (DESY), Photon Science, Hamburg, Germany; 5 Centre for Neglected Tropical Diseases, Departments of Tropical Disease Biology and Vector Biology, Liverpool School of Tropical Medicine, Liverpool, United Kingdom; 6 MRC-University of Glasgow Centre for Virus Research, Glasgow, United-Kingdom; Solena Ag, UNITED STATES OF AMERICA

## Abstract

Mosquito-borne Zika virus (ZIKV) from sub-Saharan Africa has recently gained attention due to its epidemic potential and its capacity to be highly teratogenic. To improve our knowledge on currently circulating strains of African ZIKV, we conducted protein sequence alignment and identified contemporary West Africa NS1 (NS1^CWA^) protein as a highly conserved viral protein. Comparison of NS1^CWA^ with the NS1 of the historical African ZIKV strain MR766 (NS1^MR766^), revealed seven amino acid substitutions. The effects of NS1 mutations on protein expression, virus replication, and innate immune activation were assessed in human cells using recombinant NS1 proteins and a chimeric viral clone MR766 with NS1^CWA^ replacing NS1^MR766^. Our data indicated higher secretion efficiency of NS1^CWA^ compared to NS1^MR766^ associated with a change in subcellular distribution. A chimeric MR766 virus with NS1^CWA^ instead of authentic protein displayed a greater viral replication efficiency, leading to more pronounced cell death compared to parental virus. Enhanced viral growth was associated with reduced activation of innate immunity. Our data raise questions of the importance of NS1 protein in the pathogenicity of contemporary ZIKV from sub-Saharan Africa and point to differences within viral strains of African lineage.

## Author summary

Mosquito-borne Zika virus (ZIKV) of African lineage has the potential to cause epidemics alongside a high risk of fetal pathogenicity. Improved surveillance has enabled a deeper understanding of the molecular characteristics of currently circulating viral strains from sub-Saharan Africa. A remarkable conservation of ZIKV NS1 protein has been identified between recently isolated viral strains from West Africa, with data indicating that contemporary African NS1 is secreted efficiently from human cells. The protein has been shown to enhance viral replication, associated with a reduced activation of innate immune responses. The NS1 protein might therefore play a major role in the pathogenicity of contemporary ZIKV from sub-Saharan Africa.

## Introduction

The mosquito-transmitted Zika virus (ZIKV) belonging to *Orthoflavivirus* genus of *Flaviviridae* family has become an increasingly important global health problem [1]. In the past decade, the expansion of the geographic distribution and rapid spread of the ZIKV Asian genotype has led to major epidemics in the South Pacific in 2013, and for the first time in South America in 2015 [1,2]. Cases of Guillain-Barré syndrome have been described in patients with confirmed ZIKV infection [1,2]. Intrauterine exposure to ZIKV can lead to severe neurodevelopmental defects such as microcephaly and other neurological complications [1,3–5]. Although ZIKV transmission is classically via infected female mosquitoes of the *Aedes* genus, sexual contact, blood transfusion and intrauterine transmission have been documented as alternative transmission routes [1]. Studies aiming to understand the features of emerging ZIKV were mostly carried out using epidemic strains of Asian lineage that had been first isolated in 2013 (South Pacific) and then 2015–16 (South America and Caribbean islands). As African ZIKV strains may also have epidemic potential and a high risk of fetal pathogenicity [6–9], the biological characteristics of currently circulating viral strains from sub-Saharan Africa (SSA) must be further characterized [10].

The genomic RNA of ZIKV is translated into a large polyprotein precursor that is co- and post-translationally processed into three structural proteins, capsid (C), precursor membrane (prM/M) and envelope (E) protein followed by seven nonstructural (NS) proteins NS1, NS2A, NS2B, NS3, NS4A, NS4B and NS5 [1,2]. Structural proteins C, prM, and E are required for the formation of infectious viral particles whereas NS proteins play important roles in viral RNA replication, protein processing, and virion assembly [1,2]. The NS proteins also contribute to innate immune response subversion strategies. Among ZIKV NS proteins, NS1 glycoprotein (352 amino-acid residues) exists as a membrane-associated homodimer in the endoplasmic reticulum (ER) where the protein associates with other NS proteins in viral replication complexes (RC) [11–13]. A part of a hydrophobic NS1 dimer is driven towards the cell membrane where the protein is present at the cell surface, from where it can also be released as soluble lipid-associated NS1 tetramer and hexamer contributing to pathogenesis of ZIKV infection [14–16]. The NS1 protein is considered a key protein in counteracting host innate immunity and also influences virus acquisition by mosquitoes during a blood meal [17–21].

Contemporary West African ZIKV strain ZIKV-15555 was sequenced from an individual infected in Guinea in 2018, whereas viral strains Senegal-Kedougou 2011, and Senegal-Kedougou 2015 were isolated from mosquito pools in Senegal in 2011 and 2015, respectively [6]. Analysis of their genomic RNA sequences showed full amino-acid conservation of the NS1 protein. Here, we characterized contemporary West Africa ZIKV NS1 protein (entitled hereafter NS1^CWA^) based on a comparative study with NS1 from the historical viral strain MR766-NIID (entitled hereafter NS1^MR766^), isolated from a non-human primate in Uganda in 1947. Expression of NS1^CWA^ and NS1^MR766^ proteins, which differ by seven amino-acid substitutions, was examined using recombinant NS1 proteins and a chimeric MR766 virus with NS1 protein from ZIKV-15555 replacing the original protein. Our data indicated that NS1^CWA^ is highly secreted from human cells compared to NS1^MR766^. Insertion of NS1^CWA^ in MR766^MC^ enhances viral growth and mitigates innate immune activation.

## Methods

### Cells and antibodies

Human embryonic kidney HEK-293T (CRL-1573, ATCC, VA, USA), human carcinoma epithelial lung A549 (InvivoGen, Toulouse, France), and monkey kidney normal VeroE6 (CCL-81, ATCC, VA, USA) cells were grown in Dulbecco’s modified Eagle’s medium (DMEM) growth medium (Thermo Fisher Scientific, Les Ulis, France) supplemented with heat-inactivated fetal bovine serum (FBS) (Dutscher, Strasbourg, France) and antibiotics (Dutscher, Strasbourg, France) at 37 °C. The purified mouse anti-*pan* flavivirus envelope E protein monoclonal antibody (mAb) 4G2 was provided by RD Biotech (Besançon, France). The purified humanized anti-flavivirus NS1 mAb 4G4 was a generous gift from Dr D. Watterson (University of Queensland, Australia). Rabbit anti-FLAG antibody obtained from DIAGNOMICS (Blagnac, France) was used to detect recombinant FLAG-tagged protein. Immunoblot assay on ISG was performed using anti-ISG15 and anti-IFIT1 antibody (Thermo Fisher Scientific, Les Ulis, France). Donkey IgG anti-rabbit IgG-Alexa Fluor 488, anti-mouse IgG-Alexa Fluor 594, and IgG anti-human IgG-Alexa Fluor 488 were purchased from (Thermo Fisher Scientific, Les Ulis, France). Donkey anti-mouse IgG-horseadish peroxidase (HRP)-conjugated secondary antibody were used as secondary antibodies was purchased from Abcam (Cambridge, UK).

### Production of chimeric MR766 virus with ZIKV-15555 NS1 protein

Reverse genetic approaches based on the Infectious-Subgenomic-Amplicons (ISA) method have been previously used to produce the infectious molecular clone intitled MR766^MC^ derived from African ZIKV strain MR766-NIID (Accession n°LC002520) [22]. The production of MR766^MC^ involves three synthetic amplicons Z-1^MR766^, Z-23^MR76^ and Z-4^MR766^ cloned into pUC57. The Z-1^MR766^ amplicon includes the CMV promoter immediately adjacent to the 5’NCR followed by the coding region for the structural proteins. The Z-23^MR766^ amplicon encodes the nonstructural proteins NS1 to NS4B. The Z-4^MR766^ amplicon encodes the NS5 protein followed by the 3’NCR and ended by hepatitis delta virus ribozyme and then SV40 poly(A) signal. The three amplicons were amplified by PCR from their respective plasmids using a set of specific primers so that Z-1^MR766^ and Z-23^MR766^ as well as Z-23^MR766^ and Z-4^MR766^ amplicons matched across at least 30 nucleotides (S1 Table). To generate live MR766^MC^, the three purified PCR products were transfected into HEK-293T cells using Lipofectamine 3000 and after 4 days, cell supernatant was recovered and used to infected VeroE6 cells in a first round of amplification (P1). After 5 days, P1 was recovered and amplified for a further 3 days to produce a working virus stock P2 on VeroE6 cells. Virus stock titers are determined by a standard plaque-forming assay on VeroE6 as previously described [22,23]. Infectious virus titers are expressed as plaque-forming units (PFU) per ml (PFU.mL^-1^). To generate a chimeric MR766^MC^ virus encoding the ZIKV-15555 NS1 protein, we used a Z-23^ZIKV-15555^ amplicon coding for ZIKV-15555 NS1 to NS4B proteins (Accession n° MN025403) that has been described elsewhere [10]. The fragment of Z-23^ZIKV-15555^ gene coding for NS1 protein was amplified by PCR using specific primers (S1 Table). Site-directed mutagenesis by PCR was conducted on Z-23^MR766^ gene to generate a sub-amplicon Z-23^MR766(NS2A/4B)^ gene coding for NS2A to NS4B proteins. HEK-293T cells were transfected with four PCR products amplified from Z-1^MR766^, NS1^ZIKV-15555^, Z-23^MR766(NS2A/4B)^, and Z-4^MR766^ amplicons. The extremities of NS1^ZIKV-15555^ amplicon can match with the 3’end of Z-1^MR766^ amplicon and the 5’end of Z-23^MR766(NS2A/4B)^ amplicon preserving the large open reading frame of viral polyprotein. The recovered chimeric MR766 virus with NS1^ZIKV-15555^ sequence was twice amplified on VeroE6 cells as described above, and a P2 working virus stock was used for further studies.

### Recombinant ZIKV NS1 proteins

Mammalian codon-optimized genes coding for the transmembrane domain II of ZIKV E protein acting as authentic NS1 signal peptide followed by the residues 1 to 352 of the ZIKV NS1 protein from viral strain MR766-NIID (Accession n°LC002520) or ZIKV-15555 (Accession n°MN025403) were established using *Homo sapiens* codon usage as reference. A glycine-serine spacer followed by a FLAG tag were inserted in-frame at the C-terminus of recombinant NS1 protein. Synthesis of gene sequences and cloning into *Nhe*-I and *Not*-I restriction sites of the pcDNA3.1-hygro (+) vector plasmid to generate recombinant plasmids pcDNA3/NS1^MR766^ and pcDNA3/NS1^CWA^ were performed by Genecust (Boynes, France). Site-directed mutagenesis was conducted on pcDNA3/NS1^MR766^ to introduce the two NS1 amino-acid substitutions S92P and Y286H. The resulting plasmid pcDNA3/NS1^MR766^-(P92, H286) was obtained by Genecust (Boynes, France). Plasmid sequences were verified by Sanger method. Production of endotoxin-free plasmids was performed by Genecust (Boynes, France). HEK-293 T cells were transient transfected with plasmids using Lipofectamine 3000.

### RT-qPCR

Total RNA was extracted from cells using RNeasy kit (Qiagen, Courtaboeuf, France) and reverse transcription was performed using random hexamer primers (intracellular viral RNA) and MMLV reverse transcriptase (Life Technologies). Quantitative PCR was performed on a ABI7500 Real-Time PCR System (Applied Biosystems-Life Technologies, Villebon-sur-Yvette, France). Data was normalized using 36B4 gene encoding RPL0 protein as housekeeping gene. For each single-well amplification reaction, a threshold cycle (*C*t) was calculated using the ABI7500 program (Applied Biosystems-Life Technologies, Villebon-sur-Yvette, France) in the exponential phase of amplification. Relative changes in gene expression were determined using the 2∂∂*C*t method and reported relative to the control. The primer pairs used for amplifying housekeeping 36B4, ISG and IFN-β mRNA and ZIKV genomic RNA (E gene) are listed in S1 Table.

### Immunoblot assay

Cell lysis was carried out by using RIPA lysis buffer (Sigma, Lyon, France). Proteins were separated by 4–12% SDS—PAGE and transferred into a nitrocellulose membrane. After blocking of the membrane for 1 h with 90% FBS or 5% milk in TBS-Tween, blots were incubated with primary antibody at a dilution of 1:200. Anti-mouse or anti-rabbit IgG HRP- conjugated secondary antibodies were used at 1:5000 dilution. For dot-blot assays, samples were directly loaded on a nitrocellulose membrane and then probed with the primary antibody and then anti-mouse, anti-human or anti-rabbit IgG HRP-conjugated secondary antibody. Membranes were developed with Pierce ECL Western blotting substrate (Thermo Fisher Scientific, Les Ulis, France) and exposed on an Amersham imager 680 (GE Healthcare). The signal intensity of probed protein was determined using Image J software.

### Confocal immunofluorescence assay

HEK-293T cells seeded on coverslips were fixed with 3.7% paraformaldehyde (PFA) in PBS. Permeabilization of fixed cells was performed for 4 min with nonionic detergent Triton X-100 at a final concentration of 0.1% in PBS. Cells were stained with primary FLAG antibody at dilution 1:1000 in PBS containing 1% bovine serum albumin (BSA) for 1 h at room temperature. Goat anti-rabbit or anti-human Alexa Fluor 488 IgG was used as the secondary antibody (1/1000) for 30 min in the dark. After washing, nucleus morphology was revealed by DAPI staining (final concentration 100 ng.mL-1 (-1 in index)). Image acquisition was carried out with a Zeiss LSM 710 laser scanning confocal microscope (Zeiss, Oberkochen, Germany) equipped with a X63 objective. Each *z*-stack was then processed using Amira 6.1 (FEI, Mérignac, France) to obtain a surface rendering image for one or two fluorescent markers.

### Flow cytometry assay

For flow cytometry analysis, cells were harvested after trypsinization, and fixed with 3.7% PFA in PBS at RT for 10 min. A solution of Triton X-100 (0.15%) in PBS was used to permeabilize fixed cells for 5 min at RT. After incubation of cells with a blocking solution for 10 min, ZIKV infectivity was assessed using the mouse anti-E protein mAb 4G2 (RD-Biotech, Besançon, France). A donkey anti-mouse Alexa Fluor 488 IgG at dilution 1:2000 was used as a secondary antibody. Immunostained cells were subjected to flow cytometric analysis using FACScan flow cytometer (CytoFLEX, Beckman Coulter, Brea, CA, USA). For each assay, at least 10000 cells were analyzed, and the percentage of positive cells was determined using CytExpert software (version 2.1.0.92, Beckman Coulter, Brea, CA, USA).

### Cytotoxic assay

For lactate dehydrogenase (LDH) assay, cells were seeded in 12-well culture plates. Cytotoxicity was evaluated by quantification of lactate dehydrogenase (LDH) release in cell cultures using CytoTox 96 nonradioactive cytotoxicity assay (Promega, Charbonnières-les-Bains, France) according to the manufacturer’s instructions. The absorbance of converted dye was measured at 490 nm with background subtraction at 690 nm.

### Statistical analysis

All statistical tests were carried out using GraphPad Prism version 10.1.1. Unpaired *t* test and ANOVA were used in this study.

## Results

### Expression of NS1^CWA^ protein

The sequence comparison between NS1^CWA^ and NS1^MR766^ proteins identified seven amino acid substitutions (Table 1). Mutations were distributed between the hydrophobic β-roll domain (amino-acids 1–29), the α/β Wing domain (amino-acids 38–151), connector (amino-acids 152–180), and the β-ladder domain (amino-acids 181–352) (S1 Fig). NS1^CWA^ residues P92/K146/I162/R213 were found to be unique in comparison with NS1^MR766^ as well as NS1 proteins from viral strains of Asia/America lineage (Table 1).

**Table 1:** Amino-acid changes in ZIKV NS1 protein. ZIKV strains ZIKV-15555, MR766, P6-740, H/PF/2013, BeH819015 and PVRABC59 are referenced under Genbank accession numbers MN025403, LC002520, KX377336, KJ776791, KU365778, and KX377337, respectively. Both ZIKV strains Senegal-Kedougou 2011 (SEN-11) and Senegal-Kedougou 2015 (SEN-15) are referenced under ENA accession number PRJEB39677.

NS1		ZIKV of Africa lineage				ZIKV of Asia/America lineage			
AA	domain	ZIKV-15555	SEN-2011	SEN-2015	MR766	P6-740	H/PF/2013	BeH819015	PVRABC59
21	β-roll	V	V	V	I	V	V	V	V
92	α/β Wing	P	P	P	S	S	S	S	S
146	α/β Wing	K	K	K	E	E	E	E	E
162	connector	I	I	I	V	V	V	V	V
191	β-ladder	K	K	K	R	K	K	K	K
194	β-ladder	A	A	A	A	V	V	V	V
213	β-ladder	R	R	R	K	K	K	K	K
236	β-ladder	V	V	V	V	I	I	I	I
264	β-ladder	V	V	V	V	V	M	M	M
286	β-ladder	H	H	H	Y	H	H	H	H

**Fig 1:**
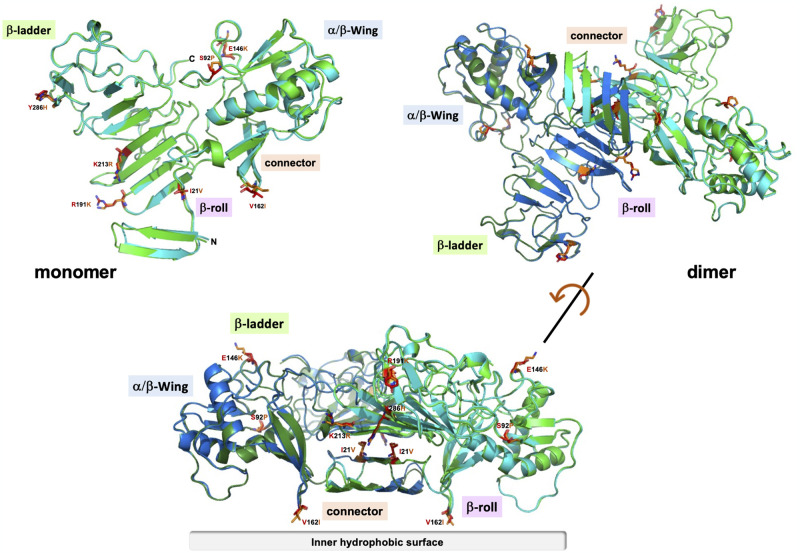
Three-dimensional structure prediction of West African ZIKV NS1 protein. AlphaFold3 was used to predict the 3D structure of NS1^CWA^ (in blue) and NS1^MR766^ (in green) monomers and dimers, which were superposed and visualized using PyMOL Molecular Graphics System. The positions of seven amino-acid substitutions that differentiate NS1^CWA^ (in orange) from NS1^MR766^ (in red) are shown in stick representation in the monomer and dimer models. The location of the different regions of the NS1 protein is indicated. In the lower part, the inner hydrophobic surface of NS1 dimer is shown [24].

AlphaFold3 was used to calculate structural models of the ZIKV NS1^CWA^ and NS1^MR766^ proteins in the monomeric and homodimeric states [24,25] (Fig 1). All predictions were characterized by high confidence, as shown by the predicted local distance test (pLDDT) scores of more than 90 for the majority of atoms (S2 Fig). Only atoms located in flexible loops and the termini of the polypeptide chains exhibited less confidence, but pLDDT values were still above 50. Moreover, the predicted template modeling (pTM) scores of 0.91 for the monomer and of 0.87 for the dimer models represented expected values for confident high-quality predictions. An interface pTM score of 0.81 was obtained for the dimer models, indicating accuracy of the predicted relative positions of the subunits within the dimeric complex. Except mutation K213R, which is located in a β-strand of the β-ladder, the other six mutations that differentiate NS1^CWA^ from NS1^MR766^ were predicted to affect amino acids located in flexible loop structures. Superposition of the models of the two NS1 monomers (RMSD 0.390 Å) and dimers (RMSD 0.264 Å) indicated no significant structural differences. This can be attributed to the multiple sequence alignment (MSA)-based structure prediction approach of AlphaFold3, which averages out the impact of individual mutations on the structural model [24,25]. However, mutations V21I, I162V, K191R, and R213K would be considered as homologous replacements. Only mutations P92S and K146E (α/β-Wing), and H286Y (β-ladder) resulted in significant changes to the spatial and electrostatic side chain properties, but these residues are all located on the protein surface with side chains pointing away from the inner structure (Fig 1). Some amino-acid substitutions might have an effect on the surface characteristics of the NS1 dimers, however, no impact is expected on the overall structure of the NS1 protein.

We first examined whether the seven amino-acid substitutions that differentiate NS1^CWA^ from NS1^MR766^ influence protein expression. Synthetic genes coding for NS1 with optimized codons for expression in human cells were inserted into an expression vector (pcDNA3). In the resulting plasmids, the sequences coding for recombinant NS1 (rNS1) proteins were preceded by the authentic signal peptide corresponding to the second transmembrane domain of adjacent E protein and ended by a short Gly-Ser spacer followed by a FLAG epitope.

**Fig 2:**
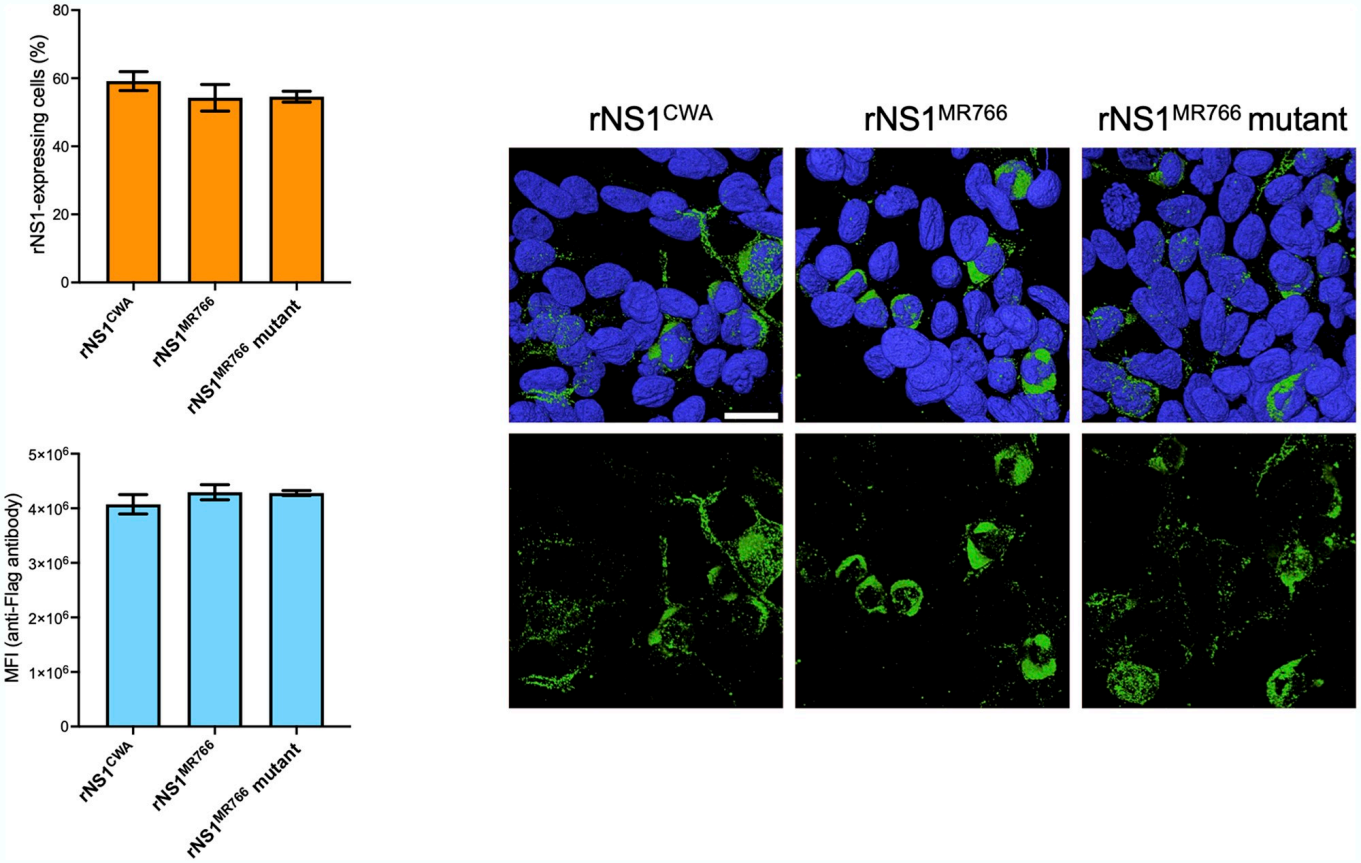
Expression osf recombinant ZIKV rNS1 proteins. HEK-293T cells were transfected with plasmids expressing rNS1^CWA^, rNS1^MR766^, or rNS1^MR766^-(P92, H286) mutant (rNS1^MR766^ mutant). *In* (**A**), FACS analysis on intracellular FLAG-tagged rNS1 proteins performed using anti-FLAG antibody. The percentage of cells positive for rNS1 expression and the mean fluorescence intensity (MFI) of FITC signal in positive cells were measured at 48 h post-transfection. The results are the mean (± SEM) of three independent assays. The differences between experimental samples are not statistically significant, using one-way Anova. *In* (**B**), three-dimensional visualization of intracellular rNS1 protein. HEK-293T cells were transfected for 24 h. Cells were stained with anti-FLAG antibody (green) as primary antibody for confocal immunofluorescence analysis. Nuclei were strained with DAPI (blue). Cell surface rendering images are shown with (top) or without (bottom) their nucleus. The same magnification was used throughout. Scale bar, 25 μM.

The NS1^CWA^-92/122 residues compose a proline-rich peptide (PRP) into the α/β Wing domain (S1B Fig). Given PRP are known to be engaged in diverse protein-protein interactions [26], site-directed mutagenesis was conducted on plasmid pcDNA3/rNS1^MR766^ to generate a mutant plasmid bearing the S92P mutation. An infrequent Tyr residue has been identified at position NS1^MR766^-286 (β-ladder) where His residue is usually found in ZIKV of different genotypes (S1 Fig and Table 1). The protonation of H286 residue might play a role in the stability of NS1 dimer [27] (Fig 1). With respect to potential involvement of H286 in pH-dependent NS1 protein stability, the Y286H mutation has been introduced in rNS1^MR766^-(S92P) mutant leading to a double mutant rNS1^MR766^-(S92P, Y286H). Expression of rNS1 proteins was verified in HEK-293T cells transfected for 48 h (Fig 2A). FACS analysis using anti-FLAG antibody showed that plasmids expressing NS1^CWA^, NS1^MR766^ or the NS1^MR766^ mutant gave a similar percentage (nearly 50%) of HEK-293T cells positive for NS1 expression (Fig 2A). The mean intensity fluorescence was comparable between the different rNS1 proteins. Thus, the ZIKV rNS1 expression constructs were suitable for further studies.

The subcellular distribution of rNS1 proteins was examined in transfected HEK-293T cells by confocal immunofluorescence microscopy using mouse anti-FLAG antibody (Fig 2B). The three-dimensional visualization of FLAG-tagged proteins showed that rNS1^MR766^ protein mostly accumulated in the perinuclear region whereas rNS1^CWA^ protein was observed to form multiple discrete foci in the cytoplasm. Thus, the rNS1^CWA^ and rNS1^MR766^ proteins can vary in their subcellular distribution in HEK-293T cells. The subcellular distribution of mutant rNS1^MR766^ protein with the (S92P, Y286H) mutations was comparable to that of rNS1^CWA^ suggesting a role for P92 and H286 residues in intracellular trafficking and/ or localization of NS1^CWA^ protein (Fig 2B).

**Fig 3:**
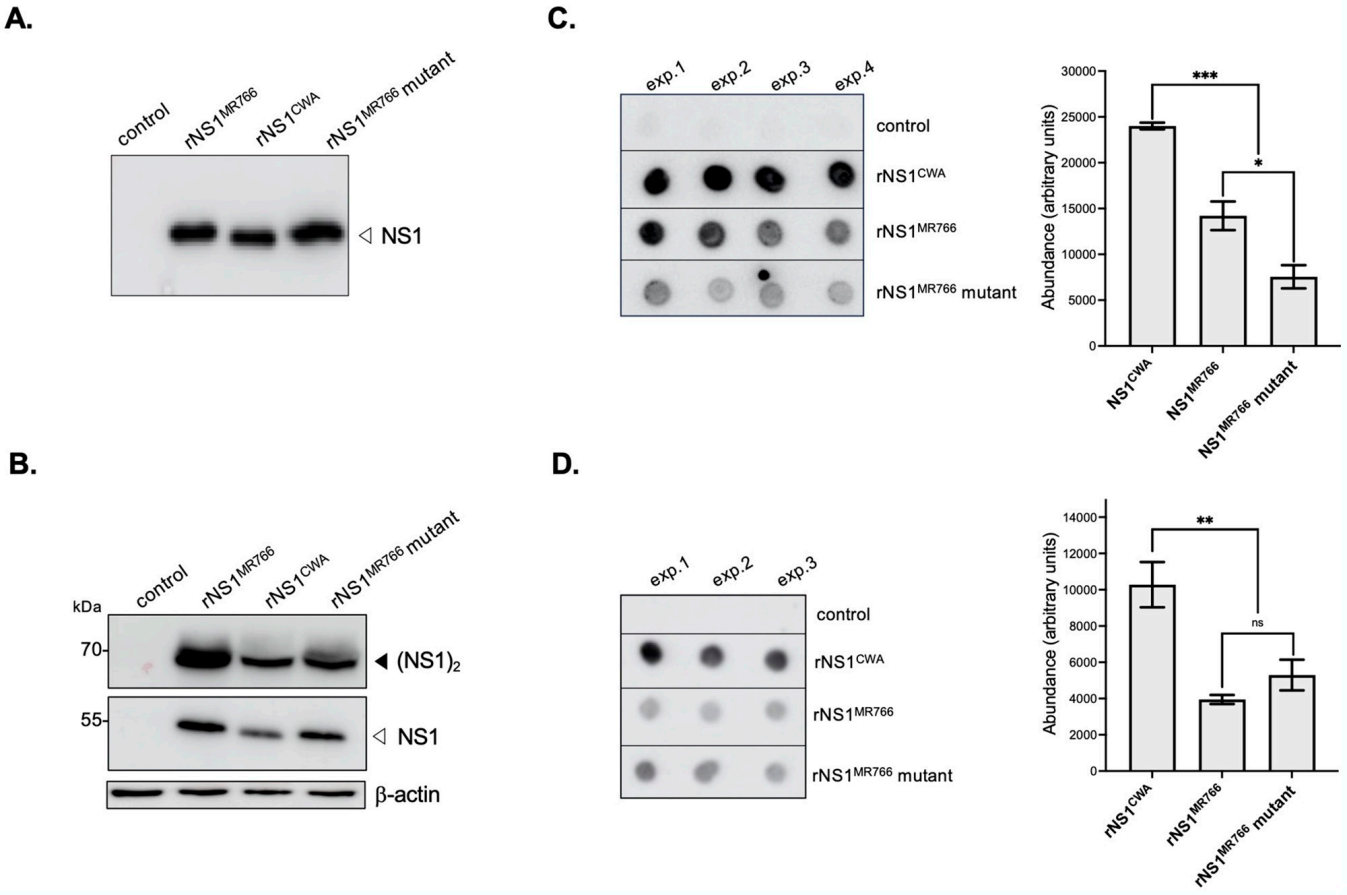
Secretion of recombinant ZIKV rNS1 proteins. HEK-293T cells were transfected for 48 h with plasmids expressing rNS1^MR766^, rNS1^CWA^, or rNS1^MR766^-(P92, H286) mutant (rNS1^MR766^ mutant), or mock-transfected (control). *In* (**A**), immunoblot assay was performed on cell lysates by probing with mouse anti-FLAG antibody. Samples were heat-denatured at 95°C for 5 min before loading on SDS-PAGE. The open head arrow indicates monomer NS1 protein. *In* (**B**), immunoblot assay was performed on cell lysates with humanized anti-NS1 mAb 4G4. Samples were heated (bottom) or not (top) before loading on SDS-PAGE. The open head arrow indicates monomer NS1 protein. The close head arrow indicates NS1 dimer. The β-actin was detected as protein-loading control for lysate samples. The close head arrow indicates NS1 dimer. *In* (**C**), cell supernatant samples from four independent transfection assays (exp.1 to exp.4) were analyzed by dot-blotting using anti-NS1 mAb 4G4. The NS1 signal intensity was quantified using Image J software to estimate the amounts of secreted soluble protein. Results are the mean (± SEM) of four replicates. Asterisks indicate that the differences between experimental samples are statistically significant, using one-way ANOVA (***p < 0.001; *p < 0.05). *In* (**D**), cell supernatant samples from three independent transfection assays were analyzed by dot-blotting using anti-FLAG antibody. The signal intensity was quantified using Image J software to estimate the amounts of secreted soluble rNS1 protein. The results are the mean (± SEM) of three replicates. Asterisks indicate that the differences between experimental samples are statistically significant, using one-way ANOVA (** *p* < 0.01; ns: not significant).

Expression of FLAG-tagged rNS1 proteins was then analyzed by immunoblot and dot-blot assays using anti-FLAG antibody or anti-NS1 mAb 4G4 (Fig 3). Given that intracellular NS1 protein essentially exists as a heat-labile homo-dimer, HEK-293T cell lysate samples were first analyzed after heating at 95°C for 5 min (Fig 3A). Anti-FLAG antibody detected comparable amounts of intracellular rNS1^CWA^, rNS1^MR766^, and rNS1^MR766^ mutant proteins, which have similar migration profiles. HEK-293T cell lysates were next assessed for the presence of dimeric forms of rNS1^CWA^, rNS1^MR766^, and rNS1^MR766^ mutant under non-denaturing conditions (Fig 3B). Anti-NS1 mAb 4G4 was able to detect dimeric forms of rNS1^CWA^, rNS1^MR766^, and rNS1^MR766^ mutant proteins (apparent MW estimated at 70 kDa). The recognition of both rNS1^CWA^ dimer and monomer by conformation-specific mAb 4G4 was lower when compared to rNS1^MR766^ (Fig 3B). Introduction of amino-acid substitutions S92P and Y286H in rNS1^MR766^ altered the reactivity of mAb 4G4, suggesting a role for the NS1-92/286 residues in the recognition or exposure of 4G4 antibody epitope.

Anti-NS1 mAb 4G4 was used to detect soluble rNS1 protein released into the extracellular environment in HEK-293T cells (Fig 3C). To estimate the secretion efficiency of rNS1, cell supernatants were collected at 48 h post-transfection and then analyzed by a dot-blot assay. A greater amount of extracellular rNS1^CWA^ protein was detected in HEK-293T cell supernatant compared with rNS1^MR766^ or rNS1^MR766^ mutant. By measuring signal intensity of dot-blot assays, we found that the amount of extracellular rNS1^CWA^ protein was at least 2-fold higher than that of rNS1^MR766^ (Fig 3C). Immunoblot assay using anti-FLAG antibody confirmed that secretion rate of rNS1^CWA^ was significantly higher in comparison to rNS1^MR766^ (Fig 3D). The presence of P92 and H286 residues was not sufficient to increase the secretion efficiency of rNS1^MR766^ protein (Fig 3C and 3D).

**Fig 4:**
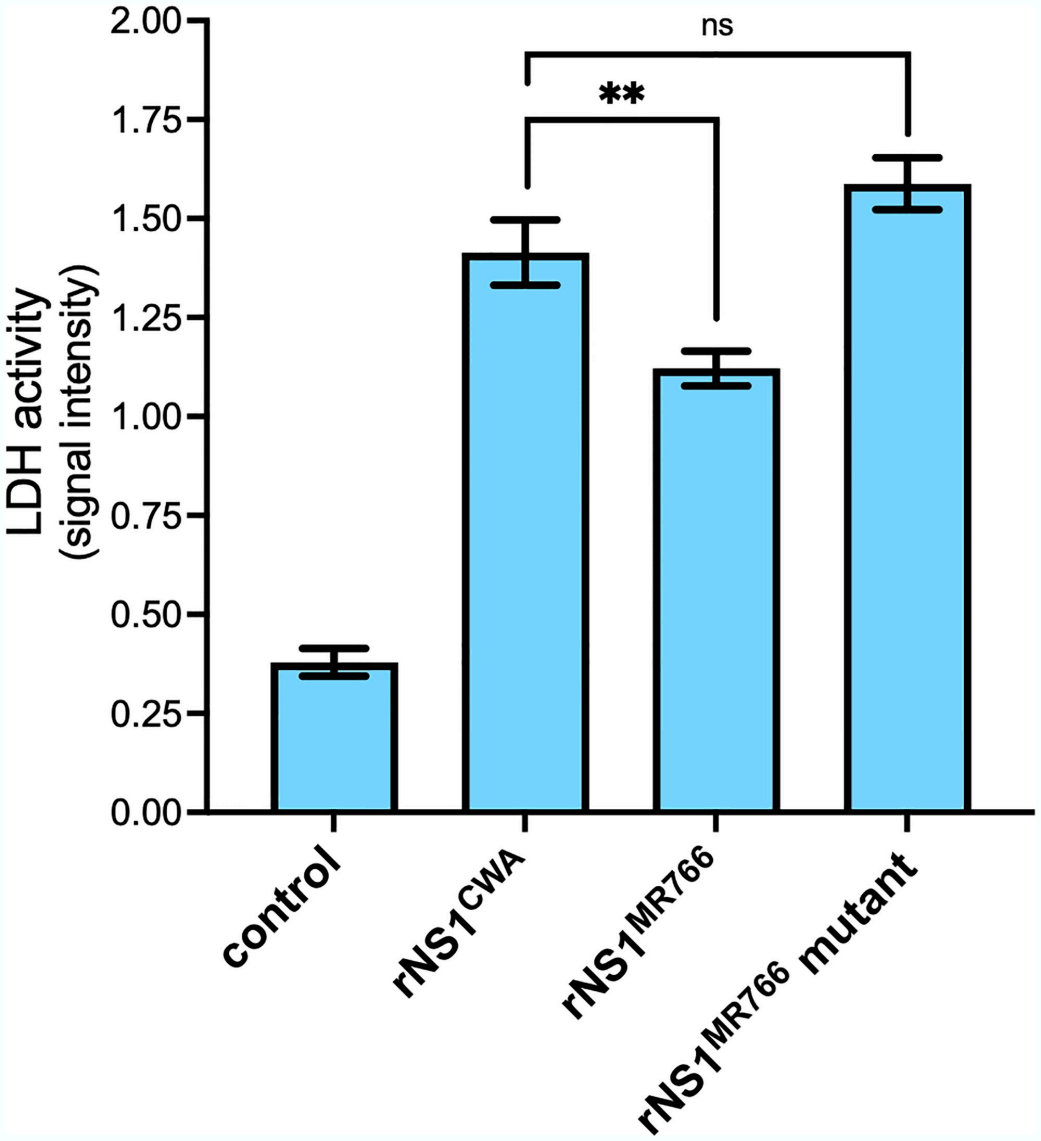
Cytotoxicity of ZIKV rNS1 proteins. HEK-293T cells were transfected for 48 h with plasmids expressing rNS1^MR766^, rNS1^CWA^, or rNS1^MR766^-(P92, H286) mutant (rNS1^MR766^ mutant), or mock-transfected (control). LDH activity was measured and O.D. values were expressed as signal intensity. The results are the mean (± SEM) of three independent assays. Asterisks indicate that the differences between experimental samples are statistically significant, using an unpaired *t* test (** *p* < 0.01; ns: not significant).

To rule out that greater amounts of extracellular rNS1^CWA^ protein reflect higher cytotoxicity of the protein, lactate dehydrogenase (LDH) activity was measured in HEK-293T cells at 48 h post-transfection (Fig 4). Although rNS1^CWA^ expression was slightly more cytotoxic than rNS1^MR766^, the effects of rNS1^CWA^ and rNS1^MR766^ mutants on cell viability were comparable despite the difference in protein secretion efficiency. Thus, it seems unlikely that secretion efficacy of rNS1^CWA^ depends on increased cytotoxicity on this cell type. Taken together, these results showed that rNS1^CWA^ and rNS1^MR766^ proteins differ in their subcellular distribution. The soluble rNS1^CWA^ protein was found in greater amounts in the cell supernatant emphasizing a role for the residues that differentiate rNS1^CWA^ from rNS1^MR766^ in the secretion efficiency of the protein. We identified a role for P92/H286 residues on rNS1^CWA^ subcellular distribution but not secretion efficiency of the protein.

### Replication of a chimeric MR76^MC^ virus with ZIKV-15555 NS1 protein

**Fig 5:**
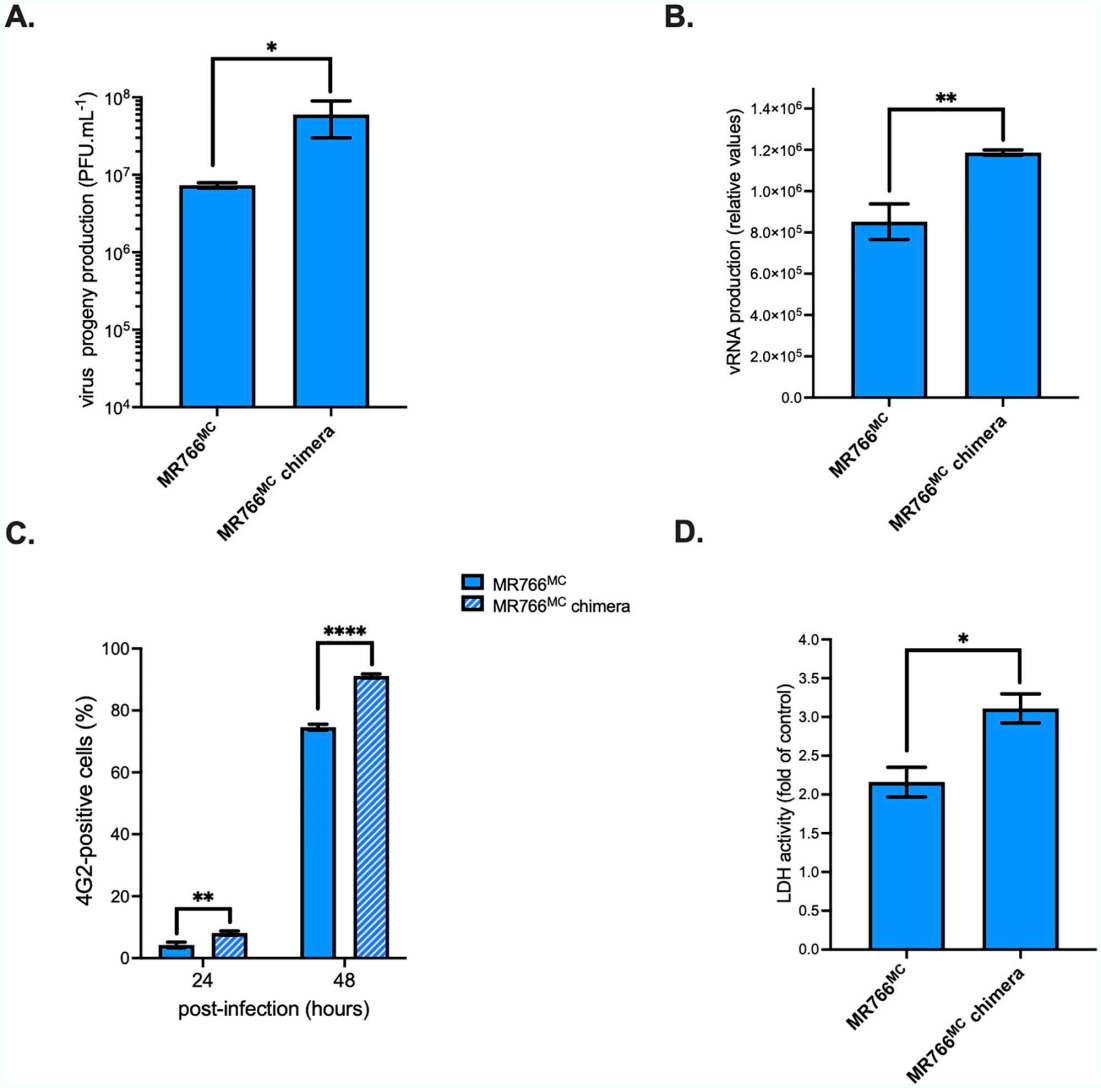
Replication of chimeric MR766^MC^ virus with NS1^ZIKV-15555^ in VeroE6 cells. Non-human VeroE6 cells were infected with MR766^MC^ or the chimeric MR766^MC^ virus with NS1^ZIKV-15555^ (MR766^MC^ chimera) at an m.o.i. of 0.1. *In* (**A**), virus progeny production (PFU.mL^-1^) was determined by plaque-forming assay. *In* (**B**), intracellular viral RNA production was determined by RT-qPCR at 48 h p.i. I*n* (**C**), the percentage of ZIKV-infected cells based on FACS analysis using anti-E mAb 4G2. *In* (**D**), LDH activity were measured at 48h p.i and expressed as a percentage relative to mock-infected cells (control). Asterisks indicate that the differences between experimental samples are statistically significant, using an unpaired *t* test (****p < 0.0001, **p < 0.01, * *p* < 0.05).

We next investigated whether the specific features of NS1^CWA^ protein influence ZIKV replication. To this end, the NS1 sequence from viral strain ZIKV-15555 (NS1^ZIKV-15555^) was introduced into infectious molecular clone MR766^MC^ from viral strain MR766-NIID by reverse genetics using the ISA method [10,22]. The resulting chimeric MR766^MC^ virus with NS1^ZIKV-15555^ instead of authentic protein includes the seven NS1 amino-acids substitutions that differentiate ZIKV-15555 from MR766^MC^. The MR766^MC^/NS1^ZIKV-15555^ chimera was first assayed for virus replication in non-human primate VeroE6 cells infected at a multiplicity of infection (m.o.i.) of 0.1 (Fig 5). The progeny production of MR766^MC^ chimera was increased by at least 10-fold at 48 h post-infection (p.i.) compared to parental virus (Fig 5A). This correlated with a vRNA rate increased by 50% (Fig 5B) and a higher percentage of ZIKV-infected cells (Fig 5C). LDH release showed that infection with chimeric MR766^MC^/NS1^ZIKV-15555^ virus caused a more pronounced loss of cell viability than parental virus (Fig 5D). Thus, chimeric MR766^MC^ virus with NS1^ZIKV-15555^ protein replicated more efficiently in non-human primate cells, leading to an increased virus progeny production associated with a more severe loss of cell viability.

**Fig 6:**
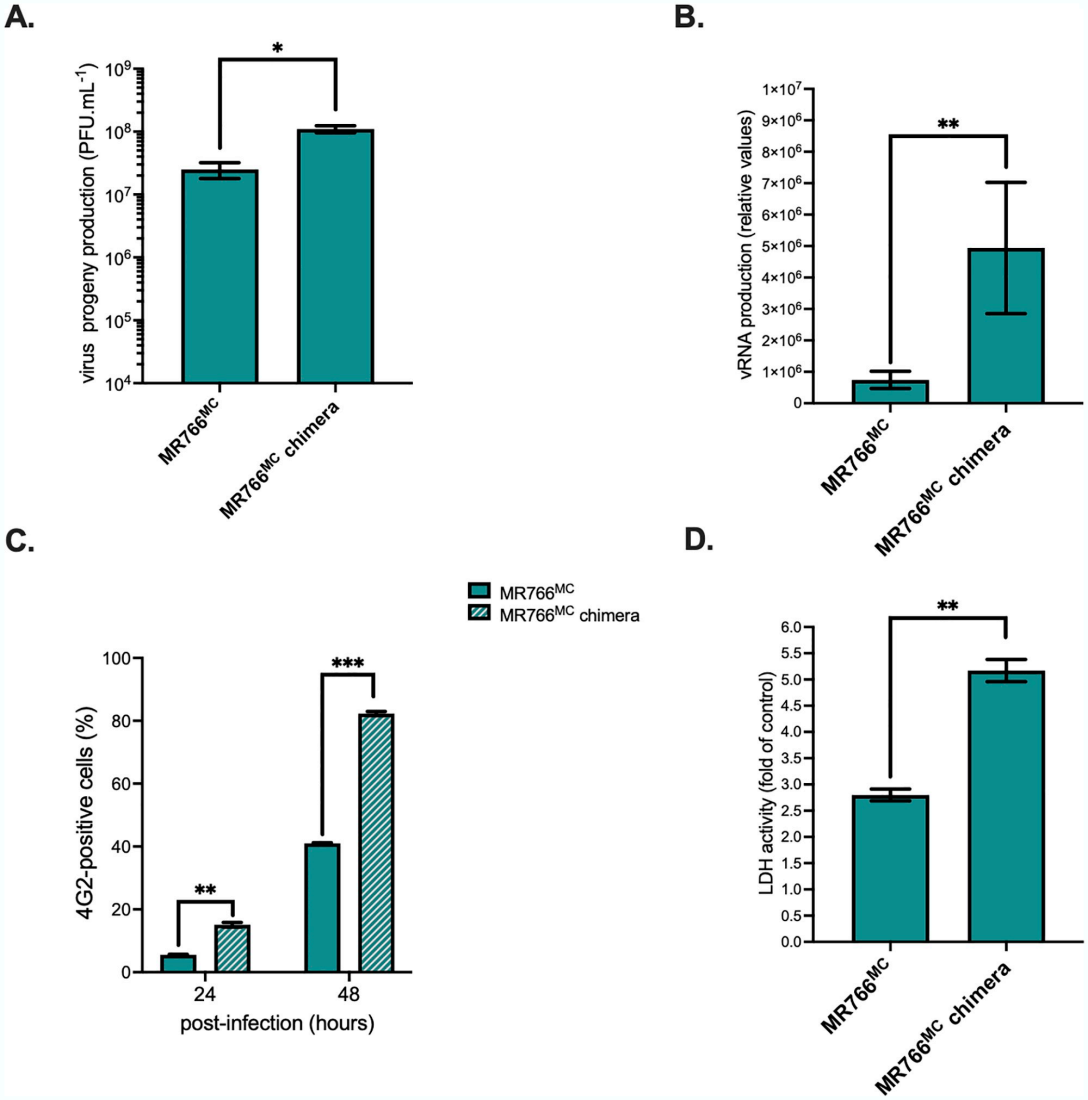
Replication of chimeric MR766^MC^ virus with NS1^ZIKV-15555^ in A549 cells. Human A549 cells were infected with MR766^MC^ or chimeric MR766^MC^ virus with NS1^CWA^ protein (MR766^MC^ chimera) at an m.o.i. of 1. *In* (**A**), virus progeny production (PFU.mL^-1^) was examined using a conventional plaque-forming assay. *In* (**B**), intracellular viral RNA production was determined by RT-qPCR at 48 h p.i. *In* (**C**), the percentage of ZIKV-infected cells based on FACS analysis using anti-E mAb 4G2. *In* (**D**), LDH activity was measured at 48h p.i and expressed as a percentage relative to mock-infected cells (control). Asterisks indicate that the differences between experimental samples are statistically significant, using an unpaired *t* test (***p < 0.001, **p < 0.01, * *p* < 0.05).

The replication efficiency of chimeric MR766^MC^ virus with NS1^ZIKV-15555^ protein was then assessed in human epithelial A549 cells which are permissive for ZIKV infection [10,23] (Fig 6). At 48 h p.i., there was an increase of nearly 1 log in progeny virus production for MR766^MC^ chimera compared to parental virus as previously observed with non-human primate cells (Fig 6A). The amount of vRNA in MR766^MC^ chimera-infected A549 cells was 10-fold higher (Fig 6B), associated to a 2-fold increase in the percentage of infected cells compared to MR766^MC^ (Fig 6C). Infection with chimeric MR766^MC^ virus with NS1^ZIKV-15555^ resulted in a pronounced loss of cell viability at 48 h p.i. (Fig 6D). Thus, a chimeric MR766^MC^ virus with NS1^ZIKV-15555^ protein displays a higher growth capacity in A549 cells compared to parental virus. Taken together, these results showed that insertion of NS1^ZIKV-15555^ protein in MR766^MC^ enhances viral replication, while inducing greater cytotoxicity. Our data indicated that the seven amino-acid substitutions that differentiate NS1^ZIKV-15555^ from NS1^MR766^ have a major effect on replication properties of ZIKV across cell lines.

### Expression of ZIKV-15555 NS1 protein

**Fig 7:**
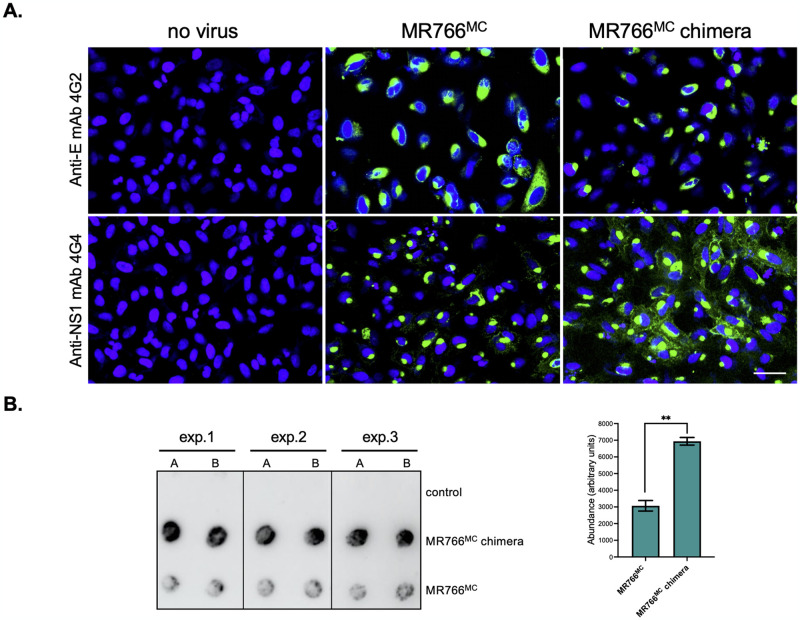
Expression of NS1^ZIKV-15555^ protein in A549 cells infected with MR766^MC^ chimera. A549 cells were infected with MR766^MC^ or chimeric MR766^MC^ virus with NS1^CWA^ protein (MR766^MC^ chimera) or mock-infected (no virus) for 24 h or 48 h at an m.o.i. of 1. *In* (**A**), visualization of intracellular rNS1 protein. Cells infected for 24h were stained with anti-E mAb 4G2 or anti-NS1 mAb 4G4 as primary antibody (green) for confocal immunofluorescence analysis. Nuclei were stained with DAPI (blue). The same magnification was used throughout. Scale bar, 25 μM. *In* (**B**), cell supernatant samples from three independent infections (exp. 1 to exp. 3) were analyzed in duplicates (A, B) by dot-blotting using mAb 4G4. The mean of signal intensity of each duplicate was determined using Image J software to estimate the relative amounts of secreted soluble NS1 protein. Results are the mean (± SEM) of three independent assays. Asterisks indicate that the differences between experimental samples are statistically significant, using an unpaired *t* test (** *p* < 0.01).

Our above results showed that insertion of NS1^ZIKV-15555^ protein in MR766^MC^ potentiates viral growth in both Vero and A549 cells. Given that NS1^ZIKV-15555^ differs from NS1^MR766^ in subcellular distribution and secretion efficiency, intracellular trafficking of NS1^ZIKV-15555^ protein from MR766^MC^ chimera was investigated in A549 cells infected for 24 h (Fig 7). Infection of A549 cells with MR766^MC^ and MR766^MC^ chimera was verified using anti-E mAb 4G2 (Fig 7A). Anti-NS1 mAb 4G4 detected intracellular NS1^ZIKV-15555^ protein as multiple in discrete foci in the cytoplasm whereas NS1^MR766^ was mostly observed in the perinuclear region (Fig 7A). Thus, the intracellular distribution of NS1^ZIKV-15555^ protein from MR766^MC^ chimera was similar to that observed with rNS1^CWA^ protein. Anti-NS1 mAb 4G4 was used to estimate the secretion level of soluble NS1^ZIKV-15555^ protein in the supernatants of A549 cells infected for 48 h with chimeric MR766^MC^ virus (Fig 7B). By dot-blot assay, we observed that infection of A549 cells with MR766^MC^ chimera resulted in the release of higher levels of NS1 protein compared to parental virus. The amount of extracellular NS1^ZIKV-15555^ was found be at least 2-fold higher than that of NS1^MR766^ (Fig 7B). Thus, the phenotype of NS1^ZIKV-15555^ expressed in A549 cells infected with MR766^MC^ chimera is similar to that observed with rNS1^CWA^ protein. Such result highlights the ability of NS1^ZIKV-15555^ to improve replication efficiency of ZIKV in line with the intracellular trafficking of the protein.

**Fig 8:**
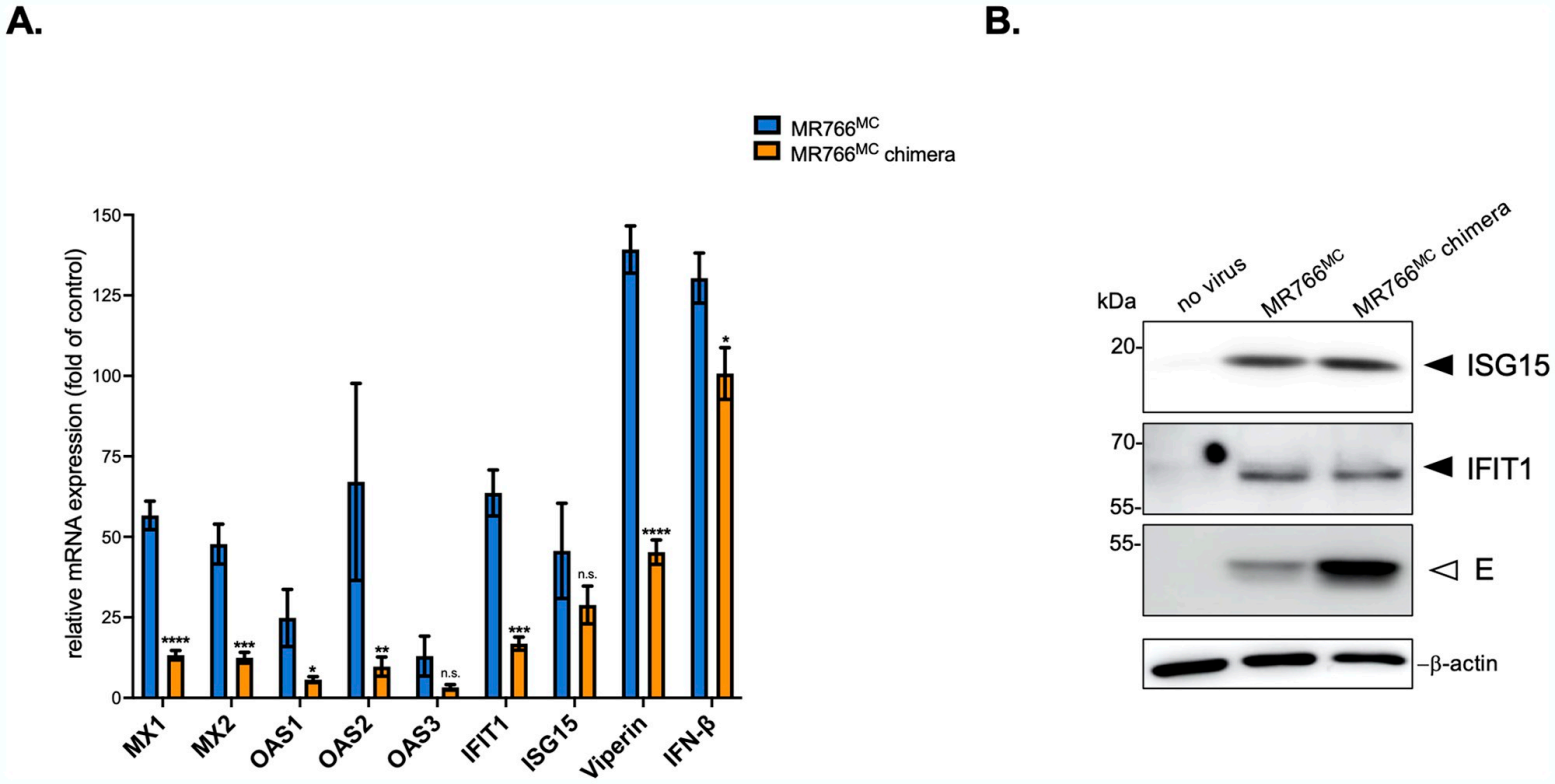
Induction of *IFN-β* and ISG expression in A549 cells infected by ZIKV. A549 cells were infected with MR766^MC^ or the chimeric MR766^MC^ with NS1^ZIKV-15555^ (MR766^MC^ chimera) at an m.o.i. of 1. *In* (**A**), the relative abundance of IFN-β and ISG mRNA was determined at 48 h p.i. by RT-qPCR. Housekeeping gene RPLPO36B4 mRNA served as an internal reference. The results are the mean (± S.D.) of three replicates. Asterisks indicate that the differences between MR766^MC^ and MR766^MC^ chimera for each cellular factor are statistically significant, using an unpaired *t* test (****p < 0.0001, ***p < 0.001, **p < 0.01, *p < 0.05). *In* (**B**), Immunoblot assay was performed on cell lysates using anti-ISG15 or anti-IFIT1 antibodies as indicated. Anti-E mAb 4G2 was used to detect ZIKV E protein. β-actin was detected as protein-loading control for lysate samples.

ZIKV infection results in activation of the intracellular signaling pathways leading to antiviral innate immune responses [28]. We next examined whether the greater vulnerability of A549 cells to chimeric MR766^MC^ virus with NS1^ZIKV-15555^ depended on ISGs and IFN-β (Fig 8). The relative abundance of IFN-β mRNA and various ISGs mRNAs was assessed by RT-qPCR on total RNA extracted from ZIKV-infected cells at 48 h p.i. (Fig 8A). Infection with MR766^MC^ induced expression of *IFN-β* and ISGs with antiviral effectors such as *Mx*, *OAS*, *IFIT*, *ISG15*, and *viperin*. Immunoblot assay with anti-IFIT1 or ISG15 antibody confirmed the upregulation of ISG expression during ZIKV infection (Fig 8B). Importantly, we observed lower transcription levels of *IFN-β* and some ISGs in A549 cells infected by chimeric MR766^MC^ virus with NS1^ZIKV-15555^ protein compared to parental virus (Fig 8A). Only *ISG15* and *OAS3* transcripts were not significantly different. Thus, NS1^ZIKV-15555^ protein can negatively regulate *IFN-β* and specific ISGs in ZIKV-infected A549 cells. The data indicated that NS1^CWA^ protein affects the activation of innate immune responses upon ZIKV infection.

## Discussion

Greater attention must be paid to recently isolated ZIKV of the African lineage, due to their epidemic potential and capacity to be highly teratogenic in humans [6]. The orthoflavivirus NS1 protein has been used or considered as a major viral target for inclusion in diagnosis, vaccine or development of antivirals [29–33]. To our knowledge, the present study is the first characterization of ZIKV NS1 protein from currently circulating viral strains from SSA. We found a remarkable conservation of NS1 amino-acid residues between recently isolated viral strains in Senegal and Guinea (referred as NS1^CWA^ (CWA in index)). We noted that NS1^CWA^ differs of NS1 protein from the historical African ZIKV strain MR766 (NS1^MR766^) by seven amino-acid substitutions (Table 1). The NS1^CWA^ protein included amino-acid residues Pro and Lys at positions 92 and 146, respectively. Whereas a short polar residue Ser is frequently found at position 92 of African and Asian/American ZIKV NS1, a hydrophobic rigid Pro residue has been identified in NS1^CWA^ protein (Table 1). The P92 residue adds to a PRP motif (amino-acids 92–112) that might play a role in regulation of signaling cascade(s) during ZIKV infection. It is worth noting that PRP is part of a region comprising the hydrophobic residues 90 to 130 that have been proposed to interact with host factors [34]. An infrequent Lys residue has been identified at position 146 where a Glutamic acid is commonly found (Table 1). The non-conserved amino-acid change Glu-to-Lys represents an important charge-changing mutation in NS1^CWA^ protein. The two non-conserved amino-acid substitutions at positions NS1-92/146 have been identified in the α/β Wing domain which shares structural homology with retinoic acid-inducible gene I (RIG-I) and melanoma differentiation-associated protein 5 (MDA5). These double-stranded RNA sensors are known to engage signaling cascades leading to type-I IFN genes activation [35–37]. The analysis of the 3D structure prediction of the NS1 dimers suggests that the amino acid replacements do not induce significant structural changes between NS1^CWA^ and NS1^MR766^ proteins. Most of amino-acid substitutions that differentiate the two NS1 are exposed toward the outer polar surface of the dimeric form (Fig 1). We cannot rule out that such mutations impact on the formation of soluble NS1 oligomers and their interactions with host partners.

With the aim of understanding the importance of mutations in the respective features of NS1^CWA^ and NS1^MR766^ proteins, a comparative analysis was conducted using recombinant NS1 proteins expressed in HEK-293T cells and a chimeric MR766 virus with NS1^CWA^ replacing the original protein. Analysis of recombinant ZIKV NS1 proteins expressed in HEK-293T cells revealed that NS1^CWA^ and NS1^MR766^ proteins can differ in their subcellular distribution. The NS1^CWA^ protein was detected in discrete foci-like structures in the cytoplasm whereas NS1^MR766^ protein mostly accumulated in the perinuclear region. Introduction of amino-acid substitutions S92P and Y286H in a recombinant NS1^MR766^ resulted to a mutant protein displaying a subcellular distribution that resembles NS1^CWA^. The reactivity of conformation-specific NS1 antibody mAb 4G4 was lower with NS1^MR766^ mutant, as compared with wild-type protein. Thus, the P92 and H286 residues can interfere with antigenic reactivity and trafficking of the ZIKV NS1 protein [38]. There was a higher secretion efficiency of NS1^CWA^ protein compared to NS1^MR766^ protein from human cells. Given that P92/H286 residues were not sufficient to increase NS1 secretion despite their impact on protein trafficking, introduction of other amino-acid substitutions in recombinant NS1^MR766^ protein could help interpret NS1^CWA^ residues in secretion efficiency of contemporary ZIKV NS1 from West Africa. Particular attention should be paid on non-conserved amino-acid change Glu-to-Lys at position NS1-246 which is exposed to the outer hydrophilic surface of the NS1 dimer (Fig 1).

Growth of chimeric MR766^MC^ virus with NS1^ZIKV-15555^ protein was enhanced, with increased virus progeny production and higher cytotoxicity compared to parental virus. The improved growth of chimeric MR766^MC^ virus was observed in both VeroE6 and A549 cells. Given the involvement of NS1 protein in viral RNA replication [12], NS1^CWA^ might have a greater propensity to interact with the other NS proteins thus enhancing activity within RCs [13]. Also, NS1^CWA^ may be more efficient in the ER re-modeling and vesicle packet formation that are required for an effective viral RNA replication [39,40]. Interestingly, the phenotype of recombinant NS1^CWA^ protein was recapitulated in A549 cells infected with a MR766^MC^ chimera expressing NS1^ZIKV-15555^ protein. Whether the biological properties of NS1^CWA^ protein make viral morphogenesis and/or virus entry easier is an important issue that requires further investigation [40]. We showed that MR766^MC^ infection induces *IFN-β* expression in A549 cells. There was a lower magnitude of *IFN-β* mRNA up-regulation in A549 cells infected by chimeric MR766^MC^ virus expressing NS1^CWA^ protein. Whether the efficiency of NS1^CWA^ protein to antagonize Type-I IFN induction contributes to efficient virus replication remains to be elucidated. IFN-β induces the expression of a large numbers of ISGs through the JAK/STAT pathway [41]. Among ISGs with antiviral functions, we found that MR766^MC^ induced *Mx*, *OAS*, *IFIT1*, *viperin* and *ISG15* mRNA expression in A549 cells. It has been reported that IFIT1 interacts with STING/MITA to negatively regulate IRF3 activation and also inhibits the mRNA translation to restrain orthoflavivirus replication [42]. Viperin has been identified as a major ISG controlling of ZIKV infection through inhibition of viral RNA translation [43–46]. Infection with MR766^MC^ chimera expressing NS1^CWA^ protein resulted in a lower magnitude of *IFIT1* and *Viperin* mRNA induction in A549 cells compared to parental virus. Insertion of NS1^CWA^ protein in MR766^MC^ caused no change in ISG15 expression consistent with the notion that ZIKV exploits ISG15 as a negative regulator of type- I IFN signaling to benefit its replication [41]. Further investigation will establish whether NS1^CWA^ protein manipulates some ISGs with antiviral functions by targeting JAK/STAT-mediated downstream events that are essential for activation of the IFN-β responsive genes.

Orthoflavivirus NS1 protein interacts with cellular factors contributing to disease pathology [16]. ZIKV NS1 protein has been shown to subvert host innate immune pathway involving RIG-I-like receptors and Toll-Like receptors [47]. We recently reported than an infectious molecular clone derived from viral strain ZIKV-15555 is efficient in preventing innate immunity activation during ZIKV infection [10]. With the purpose of expanding our current knowledge on the replication properties of contemporary African ZIKV strains, it will be necessary to investigate the effects of NS1^CWA^ protein on pattern recognition receptors [48]. Infection with ZIKV including MR766 induces inflammatory cytokines such as TNF-α, IL-6 and IL-1β [49–52]. In view of the importance of NS1 protein in activating inflammatory responses, further research into NS1^CWA^ to better understand the interactions of the protein with host cellular factors, and for example their effects on the intercellular junction complex that contributes to barrier integrity [53], will be required. Such findings will be imperative for effective Zika disease control targeting contemporary SSA viral strains.

## Supporting information

S1 Table Sequences of primers for ISA method and RT-qPCR used in this study. (DOCX)

S1 Fig ZIKV NS1 sequence alignment. *In* **(A),** NS1 protein sequences of viral strains ZIKV-1555, accession n°MN025403; SEN-11 and SEN-15 ENA accession n°PRJEB39677; MR766, accession n°LC002520; P6-740, accession n°KX377336; H/PF/2013, accession n°KJ776791; BeH819015, accession n°KU365778; PVRABC59, accession n°KX377337. The domains of NS1 protein are colored. The β-roll domain (amino-acids 1–29), the α/β Wing domain (amino-acids 38–151), the connector (amino-acids 152–180), and the β-ladder domain (amino-acids 181–352) are colored as shown. Amino-acid changes between NS1^CWA^ and MR766 are indicated in bold. *In* **(B),** alignment of residues 90–112 from NS1^CWA^ and NS1^MR766^ proteins with Pro residues in bold and underlined.
(TIFF)

S2 Fig Confidence of AlphaFold3 structure prediction. The images of the structure predictions of NS1^CWA^ and NS1^MR766^ monomers and dimers are colored by the per-atom confidence estimate pLDDT on a 0–100 scale according to the legend below. The higher the pLDDT score the higher the confidence of the atoms in the structural model. Most atoms show pLDDT scores of more than 90, indicating high confidence of the models. Only flexible loop structures and the termini of the polypeptide chains are estimated with lower confidence, however still above 50.
(TIFF)
