# Supplementary material for: The NS1 protein of contemporary West African Zika virus potentiates viral replication and reduces innate immune activation
Source: PLoS Negl Trop Dis. 2024 Aug 23;18(8):e0012146. doi: 10.1371/journal.pntd.0012146 (PMC11376516; doi:10.1371/journal.pntd.0012146)
Supplement: S1 Table — (DOCX) [file pntd.0012146.s001.docx]

S1_Table. **Sequences of primers for ISA method and RT-qPCR used in the study**

| Primer Name | Sequence (5’ to 3’) | Target Gene |
| --- | --- | --- |
| **Z1 F** | GCGGCCGCTAGTTATTAATAGTAATC | 5’end of MR766 amplicon n°1 |
| **Z1 R** | CCCCCAGGACTGCCATTCTCTTG | 3’end of MR766 amplicon n°1 |
| **Z2 F** | GAGGCCACTGTGAGAGGCGCCAAGAG | 5’end of MR766 amplicon n°23 |
| **Z3 R** | GAGAGCACTCTGAGTGTTCGTGTC | 3’end of MR766 amplicon n°23 |
| **Z4 F** | CCTGAAGTGGAAGAGACACGAAC | 5’end of MR766 amplicon n°4 |
| **Z4 R** | GCGCGCTAAGATACATTGATGAGTTTGG | 3’end of MR766 amplicon n°4 |
| **NS1 F** | CTTGGAGTGCTTGTGATTCTAC | 5’end of ZIKV-15555 amplicon n°23 |
| **NS1 R** | CCTGCACCATGAGTAGAATCAC | 3’end of ZIKV-15555 amplicon n°23 |
| **ZIKV E-F** | gatgttgtcttggaacatg | Zika virus enveloppe (E) |
| **ZIKV E-R** | tcaaggtaggcttcaccttg | Zika virus enveloppe (E) |
| **RPLPO36B4 F** | AGATGCAGCAGATCCGCAT | Ribosomal Protein Lateral Stalk Subunit P0 |
| **RPLPO36B4 R** | GGATGGCCTTGCGCA | Ribosomal Protein Lateral Stalk Subunit P0 |
| **IFNβ-F** | TGTCAACATGACCAACAAGTGTCT | Interferon Beta 1 |
| **IFNβ-R** | GCAAGTTGTAGCTCATGGAAAGAG | Interferon Beta 1 |
| **IFIT1-F** | GCGCTGGGTATGCGATCTCT | Interferon Induced Protein with Tetratricopeptide Repeats 1 |
| **IFIT1-R** | AAGCGGACAGCCTGCCTTAG | Interferon Induced Protein with Tetratricopeptide Repeats 1 |
| **ISG15-F** | TGGCGGGCAACGAATT | Interferon-Stimulated Gene 15 |
| **ISG15-R** | GGGTGATCTGCGCCTTCA | Interferon-Stimulated Gene 15 |
| **MX1-F** | CACCAGCGACAAGCGGAAGTT | MX Dynamin Like GTPase 1 |
| **MX1-R** | AGTCGTCAGTCCAGTGGCTACC | MX Dynamin Like GTPase 1 |
| **MX2-F** | GAACAATCAGCCACCACCAGGA | MX Dynamin Like GTPase 2 |
| **MX2-R** | TTCAGCACCAGCGGACACCT | MX Dynamin Like GTPase 2 |
| **OAS1-F** | GCAGACGATGAGACCGACGAT | 2'-5'-Oligoadenylate Synthetase 1 |
| **OAS1-R** | GCACTGGCATTCAGAGGATGGT | 2'-5'-Oligoadenylate Synthetase 1 |
| **OAS2-F** | TGCTCTCGGTGCTTCCAACTCA | 2'-5'-Oligoadenylate Synthetase 2 |
| **OAS2-R** | TGGCTGCTGGCATAGAGGATGT | 2'-5'-Oligoadenylate Synthetase 2 |
| **OAS3-F** | ATGCCGACCTCGTGGTGTTC | 2'-5'-Oligoadenylate Synthetase 3 |
| **OAS3-R** | AACTGCCGCTCCTGTTGACAT | 2'-5'-Oligoadenylate Synthetase 3 |
| **VP-F** | CGTGAGCATCGTGAGCAATG | Viperin |
| **VP-R** | GCTGTCACAGGAGATAGCGA | Viperin |
