# Supplementary figures and images for: The NS1 protein of contemporary West African Zika virus potentiates viral replication and reduces innate immune activation
Source: PLoS Negl Trop Dis. 2024 Aug 23;18(8):e0012146. doi: 10.1371/journal.pntd.0012146 (PMC11376516; doi:10.1371/journal.pntd.0012146)

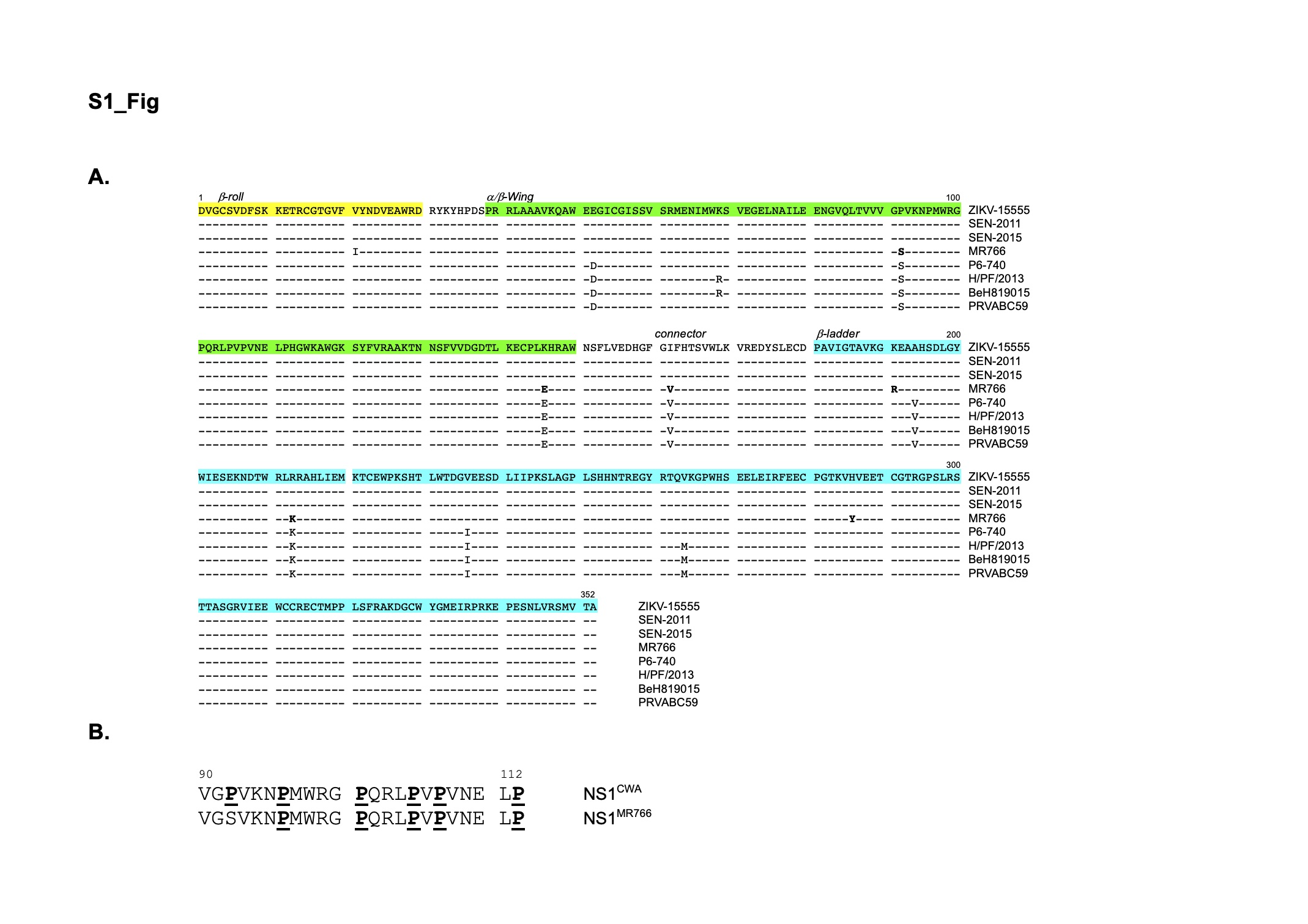

Supplement: S1 Fig — In (A), NS1 protein sequences of viral strains ZIKV-1555, accession n°MN025403; SEN-11 and SEN-15 ENA accession n°PRJEB39677; MR766, accession n°LC002520; P6-740, accession n°KX377336; H/PF/2013, accession n°KJ776791; BeH819015, accession n°KU365778; PVRABC59, accession n°KX377337. The domains of NS1 protein are colored. The β-roll domain (amino-acids 1–29), the α/β Wing domain (amino-acids 38–151), the connector (amino-acids 152–180), and the β-ladder domain (amino-acids 181–352) are colored as shown. Amino-acid changes between NS1CWA and MR766 are indicated in bold. In (B), alignment of residues 90–112 from NS1CWA and NS1MR766 proteins with Pro residues in bold and underlined. (TIFF) [file pntd.0012146.s002.tiff]

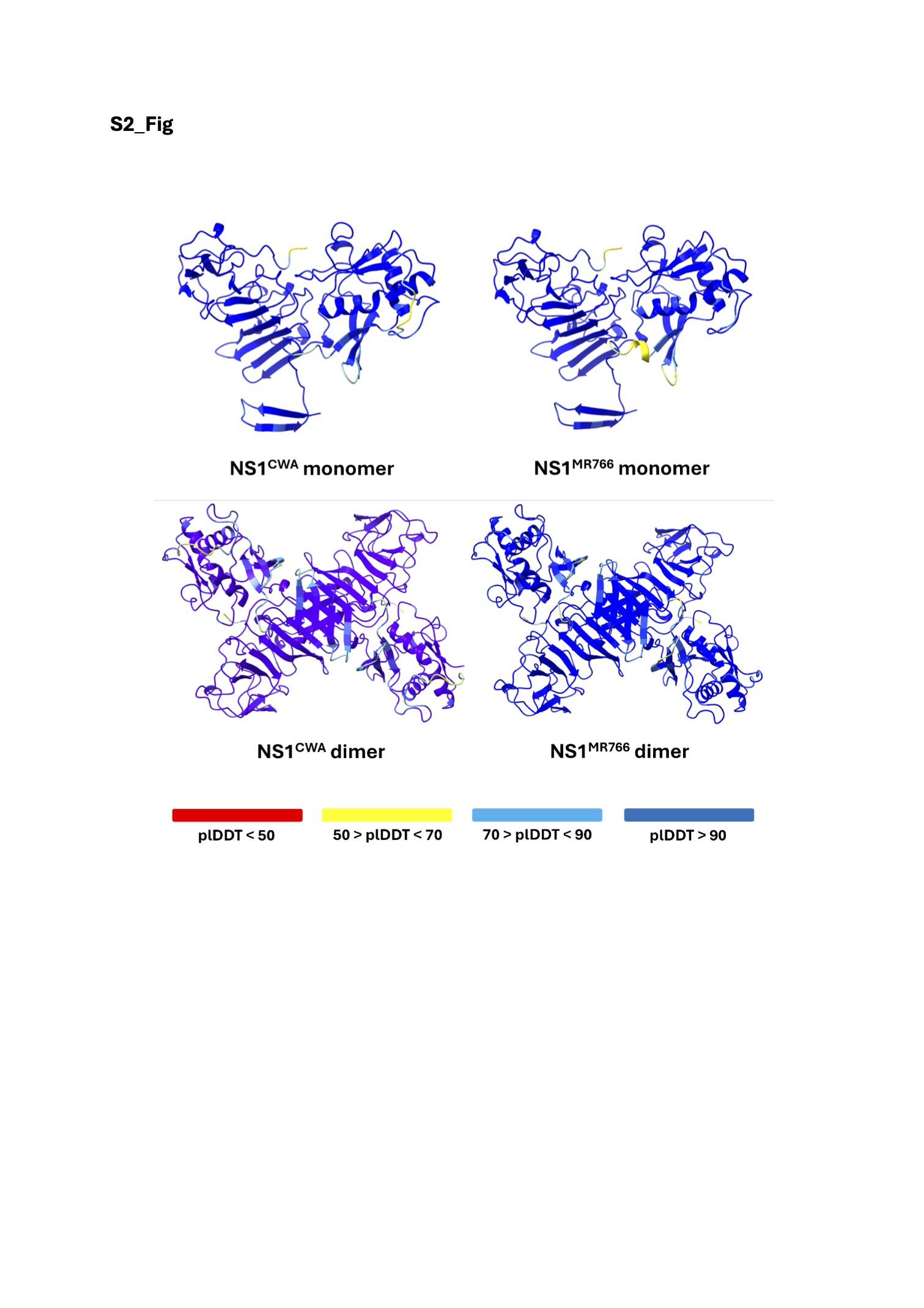

Supplement: S2 Fig — The images of the structure predictions of NS1CWA and NS1MR766 monomers and dimers are colored by the per-atom confidence estimate pLDDT on a 0–100 scale according to the legend below. The higher the pLDDT score the higher the confidence of the atoms in the structural model. Most atoms show pLDDT scores of more than 90, indicating high confidence of the models. Only flexible loop structures and the termini of the polypeptide chains are estimated with lower confidence, however still above 50. (TIFF) [file pntd.0012146.s003.tiff]
